# Sleep health associations with serum metabolites in healthy adults

**DOI:** 10.1016/j.bbih.2025.101050

**Published:** 2025-07-04

**Authors:** Charlotte Sørensen, Ilona Dudka, Ana Virel, Ingemar Kåreholt, Leonie JT. Balter, John Axelsson, Grégoria Kalpouzos, Shireen Sindi

**Affiliations:** a Division of Clinical Geriatrics, Center for Alzheimer Research, Department of Neurobiology, Care Sciences and Society, Karolinska Institutet, Stockholm, Sweden; b Department of Chemistry, Umeå University, Umeå, Sweden; c Department of Medical and Translational Biology, Umeå University, Umeå, Sweden; d Institute of Gerontology, School of Health and Welfare, Jönköping University, Sweden; e Department of Psychiatry, Radboud University Nijmegen Medical Centre, Nijmegen, Netherlands; f Donders Institute for Brain, Cognition and Behaviour, Radboud University Nijmegen, Nijmegen, Netherlands; g Department of Psychology, Stress Research Institute, Stockholm University, Stockholm, Sweden; h Department of Clinical Neuroscience, Karolinska Institutet, Stockholm, Sweden; i Aging Research Center, Department of Neurobiology, Care Sciences and Society, Karolinska Institutet and Stockholm University, Stockholm, Sweden; j Ageing Epidemiology Research Unit (AGE), School of Public Health, Faculty of Medicine, Imperial College London, London, UK

**Keywords:** Sleep timing, Karolinska sleep questionnaire, Metabolomics, Sleep health

## Abstract

**Study objectives:**

Short and long sleep duration as well as poor sleep quality have been linked to higher prevalence of metabolic disorders. However, it is still unclear how diverse sleep variables relate to different metabolic pathways. This study examines how different features of sleep health relate to serum metabolites.

**Methods:**

The study used data from 197 healthy individuals aged 20–79 (Females n = 103) from the IronAge study performed at Karolinska Institutet in Sweden. Sleep variables were assessed with the Karolinska Sleep Questionnaire, where the following variables were computed: sleep duration, sleep debt, midpoint, social jetlag (i.e., the discrepancy between midpoint on free and workdays), napping frequency and sleep quality. Morning fasting blood samples were collected and ^1^H NMR spectroscopy was utilized for metabolomic analysis. The metabolites were categorized according to their major metabolic pathways: amino acid, lipid, carbohydrate, energy and gut microbiota. Linear regressions were performed to examine the relationship between each sleep variable and metabolite.

**Results:**

Sleep duration, midpoint of sleep on free days, social jetlag and chronotype associated with eight metabolites at a significance level of *p<0.01*. Notably, midpoint associated with most metabolites spanning multiple pathways. A later midpoint was associated with higher levels of metabolites in the lipid pathway, and lower levels in the amino acid and energy pathway.

**Conclusion:**

These observations indicate that sleep timing features, *midpoint* and *social jetlag,* have a stronger relationship with morning metabolism than other sleep health dimensions. Following replication in larger samples, these complex relationships may hold potential for health promotion.

## Highlights

• Serum metabolites are associated with sleep duration and sleep timing.
• Sleep timing has the strongest association with serum metabolites.
• A later sleep midpoint associates with lipid metabolites, including higher LDL levels.
• Sleep duration is associated with branched-chain amino acids, leucine and isoleucine.

## 1 Introduction

Sleep is one of the most well-preserved behavioral processes throughout evolution, representing its essential role for our health (Humer et al., 2020). Sleep has restorative functions for the body (Allan Hobson, 2005) and it is crucial for the balance of catabolic and anabolic activities (Humer et al., 2020; Borbély et al., 2016). During daytime, catabolic activities (i.e., break-down of molecules) are dominant, whereas during sleep primarily anabolic activities take over (i.e., build-up of molecules) (Humer et al., 2020; Borbély et al., 2016). Several sleep variables (e.g., sleep loss, sleep quality and sleep apnea) are reported to associate with metabolic changes that increase the risk of metabolic syndrome (MetS) and metabolic diseases, such as type 2 diabetes and cardiovascular disease (Wenyu et al., 2011; Knutson et al., 2007; Zhang et al., 2023; Schmid et al., 2015).

Sleep research has historically focused on sleep disorders and sleep deficiency. However, sleep is multidimensional and has been conceptualized into five dimensions: duration, efficiency, timing, alertness, and satisfaction (Buysse, 2014). Sleep duration has a U-shaped association with MetS in healthy middle-aged adults, with both too little (<7h) and too much sleep (>8h) being associated with higher prevalence of MetS (Hall et al., 2008). Also, sleep timing associates with metabolic health, where bedtime variability associates with increased insulin resistance (Taylor et al., 2016). Further, alertness associates with metabolic health, where long naps (>1h) are associated with higher prevalence of diabetes (Zhao et al., 2021). Lastly, sleep satisfaction associates with MetS, where worse sleep quality increases the likelihood of developing MetS (Jennings et al., 2007). Together, these studies indicate that multiple sleep dimensions are associated with metabolic health.

Recently, metabolomics, i.e., large-scale study of metabolites, has emerged as a reliable and sensitive method allowing us to study sleep in relation to the whole peripheral system, and thereby investigating the association between sleep and metabolic health (Humer et al., 2020; Sengupta and Weljie, 2019). For sleep duration, both depriving (Davies et al., 2014) and restricting (Park et al., 2022; Bell et al., 2013) humans of sleep, are associated with the peripheral metabolism, and especially amino acids, including branched-chain amino acids (BCAAs), lipids and glucose (Davies et al., 2014; Park et al., 2022; Bell et al., 2013). However, findings are inconsistent, as some studies on habitual sleep duration found no associations with plasma metabolites (Xiao et al., 2017), while others found both short (<7h) and long (>9h) sleep duration to associate with lipid profiles (Fritz et al., 2023). Furthermore, sleep timing has been studied by investigating midpoint (Xiao et al., 2017) (i.e., the midpoint in bed), social jetlag (Sládek et al., 2023) (i.e., the discrepancy between midpoint on work and free days) and chronotype (Sládek et al., 2023) in relation to metabolomic profiles. A later midpoint is found to associate with amino acids, including BCAAs, and carbohydrate metabolites (Xiao et al., 2017), and both greater social jetlag and extreme chronotypes (i.e., both extreme morning and evening) are associated with lipid profiles of dyslipidemia (Sládek et al., 2023).

In summary, studies show that sleep has a close relationship with metabolism and likely on metabolic health. Currently, most knowledge on metabolites and sleep derives from studies on sleep duration and sleep disorders. Therefore, it is important to investigate different sleep dimensions to elucidate whether some dimensions might be more relevant for particular metabolites or metabolic pathways. Further, such knowledge could help guide future research by identifying which sleep features are most promising for intervention studies and inform healthcare professionals on the best ways to promote sleep health. Therefore, this study aimed to investigate metabolism in relation to four dimensions of sleep health (duration, timing, alertness, and satisfaction) by conducting metabolomic profiling of healthy individuals who filled out the Karolinska Sleep Questionnaire (KSQ). We examined the relationships between 51 serum metabolites and the following sleep variables: sleep duration, midpoint of sleep on workdays and free days, social jetlag, sleep debt, chronotype, napping frequency and sleep quality.

## 2 Method

### 2.1 Participants

The study sample is based on an observational cohort of healthy volunteers from the IronAge study performed at Karolinska Institutet in Stockholm, Sweden. The inclusion criteria were as follows: the participants had to be cognitively healthy, have an age of 20–80 years old, and have no history of neurological or psychiatric conditions. The cohort was previously described by Gustavsson and colleagues (Gustavsson et al., 2024). A total of 232 participants were invited, of which 35 participants were excluded: n = 15 due to not completing full protocol, n = 9 due to incidental findings on brain scan, n = 4 due to shift work, n = 5 due to having outlier values in metabolomic measures, and 2 due to missing metabolic measures. Thus, for the present analysis, 197 individuals were included.

### 2.2 Procedure

The IronAge protocol consisted of various procedures including the KSQ, blood sampling, cognitive assessment, brain MRI scans and physiological measures. In the present study we focus on metabolites measured in serum and self-reported sleep using the KSQ. Additional variables used for descriptive purposes include Mini Mental State Examination (MMSE) (Folstein et al., 1975) and Life's Simple 7 (LS7) (Thacker et al., 2014). All participants provided written informed consent prior to data collection, and the study was approved by the regional ethical review board in Stockholm (2016/457-31/2).

### 2.3 Computation of sleep variables using the KSQ

Different sleep variables were computed using the KSQ, a validated questionnaire to measure self-reported sleep (Nordin et al., 2013). The KSQ evaluates both nocturnal sleep disturbances and daytime sleepiness, together with questions on sleep duration, sleep window, one's own perception of sleep hours needed, and lastly it includes questions on napping frequency and chronotype. Using the KSQ, the following sleep variables were computed (Table 1).

**Table 1:** Sleep variables created based on the Karolinska Sleep Questionnaire.

Sleep variable	Measure type
Sleep duration	Continuous
Midpoint (MSF & MSW)	Continuous: Midpoint in bed compared to midnight
Social jetlag	Continuous: Midpoint on leisure days compared to working days
Sleep debt^a^	Continuous: Sleep duration compared to sleep need
Chronotype	Ordinal: *Are you an evening or a morning type of person?* 1: Definitely Early 2: Rather Early 3: Neither 4: Rather Late 5: Definitely Late
Napping frequency	Ordinal: *How often do you take naps?* 1: Never 2: Once Per Month 3: Once Per Week 4: Several Times Per Week 5: Everyday
Sleep quality index	Interval: Summation of 4 ordinal measures

a Sleep debt: The sleep need is based on the KSQ question: *“How much sleep do you need*. MSW = Mid-sleep on workdays, MSF = Mid-sleep on free days.

#### 2.3.1 Sleep duration

Sleep duration was calculated as the time spent in bed minus the time to fall asleep. First, the time between *“At which time do you normally go to sleep?”* and *“At which time do you normally wake up?”*, was calculated, and then the time “*how long time are you awake before you fall asleep*” was deducted. Participants were asked both for work and free days. First, sleep duration was calculated separately for work and free days and second a weighted average was calculated (5xsleep duration workday+2xsleep duration free day)/7). Sleep duration is known to have a U-shaped association with increased odds of various diseases (Zhu et al., 2021), thus statistical modeling of sleep duration included a quadratic term. For the analyses, the weighted average sleep duration was centered around the mean sleep duration (7.7 h) of the cohort.

#### 2.3.2 Midpoint

Midpoint of sleep is a measure of habitual chronotype based on actual sleep timing. It was determined similarly to a previous study (Xiao et al., 2017), by calculating the time difference of the midpoint from midnight. With the current calculation, a higher midpoint value indicates a later midpoint in bed. Midpoint has measures for work and free days and will be named consistently with (Roenneberg et al., 2007; Zavada et al., 2005): Mid-sleep on workdays (MSW) and mid-sleep on free days (MSF) (Table 1).

#### 2.3.3 Social jetlag

Social jetlag (Roenneberg et al., 2007; Wittmann et al., 2006) was computed by calculating the difference between MSW and MSF, such that higher values represent later MSF than MSW and vice versa (Table 1).

#### 2.3.4 Sleep debt

Sleep debt was calculated by comparing the calculated sleep duration in comparison to the question *“How much sleep do you need?”.* Sleep duration was subtracted from habitual sleep need, indicating higher sleep debt upon higher value (Table 1).

#### 2.3.5 Chronotype and napping

Chronotype and napping were based on the ordinal question in the KSQ: “*How often do you take naps?”* and *“Are you an evening or a morning type person?”*, where a higher value indicated increased napping frequency and later chronotype, respectively (Table 1).

#### 2.3.6 Sleep quality index

The KSQ consists of 18 ordinal items which can yield four sleep indexes, including a sleep quality index (Nordin et al., 2013). The summation of four questions; KSQa (“*Difficulties falling asleep*”), KSQc (“*Repeated awakenings (with difficulties going back to sleep)”),* KSQi (“*Premature (final) awakening*”) and KSQj (“*Disturbed/restless sleep*”) defines sleep quality (Nordin et al., 2013). Each question was answered in the ordinal range from 1 to 6, where a higher value indicates better sleep. Therefore, the theoretical range for sleep quality is 4–24, where higher values indicate better sleep quality.

### 2.4 Blood sample collection

Blood samples were collected by venipuncture between 8 and 10 a.m. under fasting conditions. Collected blood samples were incubated for 30 min at room temperature and then centrifuged (2000×*g*, 10 min). They were then separated into aliquots of 225 mL, frozen and stored at Karolinska Institutet Biobank (REMP300 -80 °C). Samples were transferred to Swedish NMR Center (SciLifeLab) in Umeå, Sweden for metabolomic analysis. Serum samples were stored at a temperature of −80 °C until the NMR measurements were performed.

### 2.5 ^1^H NMR analysis of serum samples

Prior to nuclear magnetic resonance (NMR) analysis, serum samples were thawed, and 125 μL aliquots were mixed with 75 μL of a buffer solution (NaH_2_PO_4_ and K_2_HPO_4_, including 0.1 % TMSP, pH 7.47) and placed into the 3 mm NMR tubes with application Gilson preparation robot (Middleton, USA). Quality control samples were prepared by pooling all samples to monitor analytical variability of the metabolic profiling platform.

The ^1^H NMR spectra were acquired using a Bruker 600 MHz AVANCE III spectrometer equipped with a TCI cryoprobe and an NMR CASE sample changer at 310.0 K. One-dimensional (1D) spectra were recorded using Carr-Purcell-Meiboom-Gill (CPMG) sequence to have enhanced visualization of low molecular weight compounds. The spectrum was acquired with a recycle delay of 4 s, 12-kHz spectral width, 73,728 data points, 30 ms total spin-echo time, total 64 scans, and 4 dummy scans. The acquired NMR spectra were manually corrected for the phase and the baseline with TopSpin 2.1 (Bruker Biospin, Germany). All chemical shifts were referenced to the signal of TMSP.

^1^H NMR spectra were aligned using icoshift 1.2 and manual integration of peaks was performed to a linear baseline on all spectra in parallel using an in-house developed Matlab routine as applied before (Chachaj et al., 2020). The integrated data were normalized to the total sum of the spectrum to give the same total integration value for each spectrum. Metabolite identification was carried out in the Chenomx NMR suite professional (version 7.72, Chenomx, Inc., Edmonton, Canada) and with application of the Human Metabolome Database (HMDB). All 51 identified metabolites are listed in supplementary material, Table S1. Prior to further analysis, unsupervised principal component analysis (PCA) was applied (Simca 17.0, Umetrics, Umeå, Sweden) to check data homogeneity, and to identify outliers based on samples’ metabolic similarities and dissimilarities.

### 2.6 Statistical analysis

Associations between metabolite levels and sleep variables were examined using a series of robust linear regressions. Each sleep variable (n = 8, Table 1) was modeled against each metabolite. All regression models were adjusted for age, sex and Body Mass Index (BMI) as these factors are associated with sleep and serum metabolite levels (Wenyu et al., 2011; Zhang et al., 2023). To account for the high number of models analyzed, we adapted to a more conservative significance level of *0.01*. Lastly, pairwise correlations between the sleep variables and serum metabolites were determined for those that survived the significance level of *p<0.01*. Spearman correlation coefficients were used to determine correlations between sleep variables due to one sleep variable being an ordinal measure. Pearson correlation coefficients were used between serum metabolites due to being continuous and normalized measures. The robust linear regressions were conducted in R (Version: 4.4.0) using the function lmrob() in the robustbase R Package (Basic and Statistics, 2024). Robust regression with lmrob() reduces the influence of outliers and provides a more robust fit (Basic and Statistics, 2024). Results are presented as beta coefficients, 95 % confidence interval (CI) and *p*-values.

## 3 Results

### 3.1 Participants

**Fig. 1:**
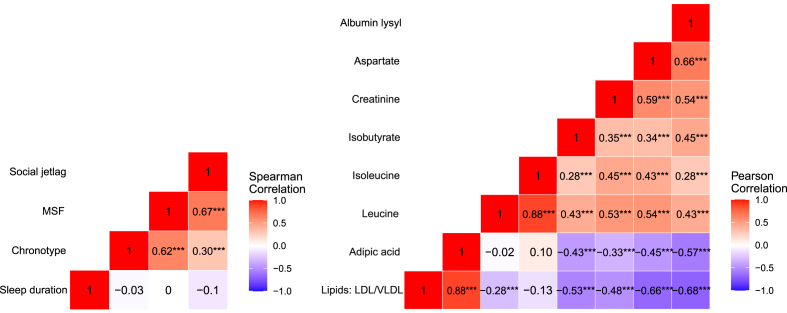
Heatmap of pairwise correlations among sleep variables and serum metabolites significant at p < 0.01. From the series of robust linear regressions, four sleep variables (sleep duration, MSF, social jetlag and chronotype) were associated with 8 metabolites at a significance level of p < 0.01. Left) Spearman correlation coefficients between the sleep variables. Right) Pearson correlation coefficients between the metabolites associated with sleep variables at p < 0.01. Pairwise correlations significant at p < 0.05∗, p < 0.01∗∗, p < 0.001∗∗∗. MSF = Mid-sleep on free days.

The characteristics of the IronAge cohort are shown in Table 2. Participants had a uniform age distribution ranging from 20 to 79 years of age. The participants reported a weighted average of 7.7 h sleep duration, a MSW of 2.8 and MSF of 3.8 yielding a 1-h social jetlag (Table 2). Sleep duration had no significant pairwise correlation with neither chronotype, MSF, nor social jetlag, whereas MSF, social jetlag and chronotype had moderate to strong positive pairwise correlations (Fig. 1).

**Table 2:** Characteristics of study participants in the IronAge analysis cohort.

Characteristics	Total	Female	Male
Total cohort Sample N	197	103	94
Age, N *(%)*			
20 to <40 years	62 (31)	32 (31)	30 (32)
40 to <60 years	62 (31)	34 (33)	28 (30)
60–79 years	73 (37)	37 (36)	36 (38)
BMI, *Mean (SD)*	24.4 (3.1)	23.7 (3.2)	25.2 (2.8)
Years of Education, M*ean (SD)*	16.1 (3.3)	16.1 (3.5)	16.1 (3.2)
MMSE, M*ean (SD)*	28.5 (1.3)	28.6 (1.3)	28.4 (1.3)
Occupation, *N(%)*			
Unemployed or retired	60 (30)	29 (28)	31 (33)
Part-time work	35 (18)	20 (19)	15 (16)
Full time work	102 (52)	54 (52)	48 (51)
Life's Simple 7, *Mean (SD)*	10.2 (1.8)	10.6 (1.8)	9.9 (1.7)
Sleep duration, *Mean (SD) [min – max]*	7.7 (0.8) [5.1–9.9]	7.8 (0.8) [5.1–9.7]	7.7 (0.8) [5.3–9.9]
Sleep debt, *Mean (SD)*	−0.1 (1.0)	−0.1 (1.0)	−0.2 (0.9)
Social jetlag, *Mean (SD)*	1.0 (0.9)	1.1 (1.0)	0.9 (0.8)
Midpoint, *Mean (SD)*			
MSW	2.8 (0.8)	2.8 (0.8)	2.9 (0.8)
MSF	3.8 (1.1)	3.9 (1.1)	3.8 (1.1)
Chronotype, N *(%)*			
Definitely early (1)	19 (9.6)	11 (11)	8 (8.5)
Rather early (2)	42 (21)	26 (25)	16 (17)
Neither (3)	55 (28)	23 (22)	32 (34)
Rather late (4)	55 (28)	27 (26)	28 (30)
Definitely late (5)	26 (13)	16 (16)	10 (11)
Napping frequency, N *(%)*			
Never (1)	76 (39)	41 (40)	35 (38)
Once per month (2)	58 (29)	31 (30)	27 (29)
Once per week (3)	34 (17)	15 (15)	19 (20)
Several times per week (4)	20 (10)	13 (12)	8 (8.5)
Every day (5)	9 (4.6)	4 (3.9)	5 (5.3)
Sleep quality Index, *Mean (SD)*	18.3 (3.4)	17.7 (3.4)	19.1 (3.2)

Descriptives are reported as proportions: N (%) for categorical and ordinal data, and for continuous data: Mean (SD) are reported. Life's simple 7 range from 0 to 14. The sleep quality index ranges from 4 to 24 where higher indicate better sleep quality. Sleep duration, sleep debt, social jetlag and midpoint all have the unit H [hour]. BMI = Body Mass Index (unit; *weight/height*^*2*^), MMSE = Mini Mental State Examination. MSW = Mid-sleep on workdays, MSF = Mid-sleep on free days.

### 3.2 ^1^H NMR analysis of serum samples

A total of 51 metabolites were identified using ^1^H NMR spectroscopy and were included in the analysis (Table S1 for full metabolite list). We identified several amino acids, glycolytic intermediates and signals from lipids among other metabolites. Data quality was assessed using principal component analysis (data not shown) and based on these results five subjects were identified as outliers and excluded from subsequent analysis. From 51 identified metabolites 8 metabolites had associations with at least one sleep variable at a significance level of p < 0.01 and 27 metabolites had associations with at least one sleep variable at a significance level of p < 0.05, and (Table 3, Fig. S1).

**Table 3:** Significant associations between sleep variables and serum metabolites.

	Sleep duration (quadratic)	MSF	MSW	Social jetlag	Chronotype	Sleep debt	Napping frequency
**Metabolites**	β ; 95 % CI ; p	β ; 95 % CI ; p	β ; 95 % CI ; p	β ; 95 % CI ;p	β ; 95 % CI ; p	β ; 95 % CI ; p	β ; 95 % CI ; p

**Within major pathway: Amino acid pathway**							

Alanine	**-**	**-**	**-**	–	–	–	−0.019;−0.034:-0.004;0.015
Leucine	**−0.014;-0.024:-0.003;0.010**	**-**	–	–	–	–	–
Isoleucine	**−0.003;-0.004:-0.001;0.000**	**-**	–	–	–	–	–
Serine	**-**	**-**	–	−0.017;−0.034:0.000;0.047	–	–	–
Aspartate	**-**	**−0.011;-0.019:-0.003;0.010**	–	**−0.016;-0.027:-0.005;0.005**	–	–	–
Threonine	**-**	0.018; 0.001:0.035; 0.036	–	0.023; 0.001:0.045; 0.045	0.017; 0.000:0.033;0.048	–	–
Formate	**-**	−0.002;−0.003:0.000; 0.024	–	−0.002;−0.004:0.000;0.026	−0.001; −0.002:0.000;0.043	–	–
Ornithine	**-**	−0.003;−0.005:0.000; 0.028	–	–	–	–	–
Glutamine	**-**	−0.027;−0.054:-0.001;0.045	–	−0.038;−0.068:-0.008;0.015	–	–	–
Glutamate	**-**	**-**	–	–	–	–	−0.005;−0.008:-0.001;0.015
Glutathione	**-**	**-**	–	−0.002; −0.004:0.000; 0.028	–	–	–
1-methylhistidine	**-**	**-**	–	−0.002; −0.005:0.000; 0.030	–	–	–
3-hydroxybutyrate	**-**	**-**	–	–	–	0.009;0.001:0.018;0.029	–

**Within major pathway: Lipid pathway**							

Lipids: LDL/VLDL	**-**	**0.831;0.238:1.424;0.006**	–	0.773; 0.129:1.417; 0.019	0.554; 0.022:1.087; 0.041	–	–
Lipids: CH_2_-C=O	**-**	0.082;0.016:0.148;0.015	–	0.093; 0.010:0.176; 0.028	0.079;0.013:0.144;0.019	–	–
Lipids: CH_2_-C=C	**-**	–	–	–	–	–	0.030; 0.004:0.055;0.023
Adipic acid	**-**	**0.118;0.029:0.208;0.010**	–	0.136; 0.016:0.257; 0.027	0.109; 0.021:0.197;0.015	–	–
Glycerol of lipids	**-**	0.013;0.002:0.025;0.021	–	–	0.014; 0.003:0.025;0.013	–	–
Albumin lysyl	**-**	**−0.008;-0.013:-0.003;0.004**	–	**−0.010;-0.018:-0.003;0.005**	–	–	–

**Within major pathway: Carbohydrate pathway**							

α-Glucose	–	−0.042;−0.076:-0.008;0.015	–	–	–	–	–
α& β-Glucose	–	−0.103;−0.186:-0.021;0.014	–	−0.101;−0.198:-0.005;0.040	–	–	–
Lactate	**-**	–	–	–	–	−0.050;−0.096:-0.004;0.035	−0.040;−0.076:-0.004;0.030
Myo-Inositol	−0.003;−0.005:0.000;0.020	–	–	–	–	–	–
Scyllo-inositol	–	–	–	−0.035;−0.067:−0.003;0.033	–	–	–

**Within major pathway: Energy pathway**							

Creatinine	−0.002;−0.003:0.000;0.011	**−0.003;-0.005:-0.001;0.010**	–	–	–	–	–
Citrate	−0.003;−0.005:0.000;0.047	–	−0.006;−0.011:-0.001;0.029	–	–	–	−0.003;−0.007:0.000;0.040

**Within major pathway: Gut microbiota pathway**							

Isobutyrate	**-**	−0.002;−0.003:0.000;0.039	−0.002; −0.003:0.000;0.038	–	**−0.002;-0.002:-0.001;0.002**	–	–

All results are significant at p < 0.05, and the results in bold are significant a p < 0.01. – indicate a non-significant model *(p>0.05).* The sleep quality index did not have significant associations with any metabolites. Robust linear regressions controlled for age, sex and BMI. MSW = Mid-sleep on workdays, MSF = Mid-sleep on free days.

#### 3.2.1 Associations between sleep duration and serum metabolites

Sleep duration, given an inverted U-shaped representation, was associated with morning levels of leucine and isoleucine at a significance level of p < 0.01 (Table 3), where leucine and isoleucine had a strong positive correlation with each other (Fig. 1). Additionally, sleep duration was associated with myo-inositol, creatinine and citrate, but these associations did not meet the p < 0.01 significance threshold (Table 3).

#### 3.2.2 Associations between midpoint and serum metabolites

A later MSF was associated with lower levels of aspartate, creatinine and albumin lysyl, and higher levels of adipic acids and lipid signal (lipids: LDL/VLDL) at a significance level of p < 0.01 (Table 3). Aspartate, creatinine and albumin lysyl had moderate positive correlations, and adipic acid and lipid signal (Lipids: LDL/VLDL) had a strong positive correlation (Fig. 1). Moreover, a later MSF was associated with more metabolites belonging to the amino acid, lipid, carbohydrate and gut microbiota pathway, however the associations did not pass the significance threshold of p < 0.01 (Table 3).

#### 3.2.3 Association between social jetlag and serum metabolites

More social jetlag was associated with lower levels of aspartate and albumin lysyl at p < 0.01 (Table 3), and aspartate and albumin lysyl had a moderate positive correlation (Fig. 1). Further, more social jetlag was associated with lower levels of metabolites in the amino acid and carbohydrate pathway and with higher levels of metabolites in the lipid pathway, but the associations did not survive the p < 0.01 significance level (Table 3).

#### 3.2.4 Association between chronotype and serum metabolites

A later chronotype was associated with lower levels of isobutyrate at p < 0.01. Additionally, a later chronotype was associated with higher lipid signals (LDL/VLDL and -CH_2_-C=O), adipic acids, threonine, and lower levels of several metabolites in the amino acid pathway; however, these associations did not meet the p < 0.01 significance threshold (Table 3).

## 4 Discussion

In this study, we investigated the associations between habitual sleep and serum metabolites in healthy individuals. Analyses indicated associations between sleep and metabolites, notably for MSF and social jetlag which had relations with metabolites in multiple metabolic pathways, including the amino acid and lipid pathway. Compared to most previous studies, we investigated sleep and metabolomics from the perspective of sleep health, adding important new insights into understanding which habitual sleep features are associated with serum metabolites in healthy individuals without sleep disorders. These findings suggest that sleep timing has a stronger relation to serum metabolites than other sleep health dimensions.

We found that both short and long sleep duration were associated with lower levels of two BCAAs, leucine and isoleucine. Our results are partly in line with previous studies where experimental sleep deprivation and sleep restriction showed effects on amino acid (Davies et al., 2014; Park et al., 2022; Bell et al., 2013). However, they observed an association between shorter sleep duration and increased levels of BCAAs, which is in the opposite direction of our results. Increased BCAAs levels were proposed to reflect a catabolic effect of restricted/deprived sleep (Park et al., 2022; Bell et al., 2013). During prolonged wakefulness there is an increased demand for energy, and this could be reflected in the change in amino acid metabolism, and especially the increase in BCAAs (Park et al., 2022; Gou et al., 2017). Thus, restricting sleep may shift the body into a catabolic state compromising the restorative function of sleep (Humer et al., 2020), which may lead to metabolic signatures associated with pathologies such as insulin resistance (Yoon, 2016). BCAAs are essential amino acids, and increasing their levels through for example BCAA supplements has also been shown to have positive effects on, for example, glucose homeostasis (Yoon, 2016). Therefore, the discrepancies between previous studies showing increased BCAA levels upon short sleep and our findings of lower BCAA levels may be attributable to the difference in sleep duration. Compared to previous experimental studies that restricted sleep (4 h/night (Park et al., 2022) and 5.5h/night (Bell et al., 2013)), our study is based on an observational cohort where sleep duration predominantly lies in the recommended range for younger to older adults (Hirshkowitz et al., 2015) (Fig. S2). Therefore, it might be that the negative effects of BCAA become apparent only at more extreme sleep restriction. In contrast, our study may indicate the beneficial effect of higher BCAA levels for a sleep duration of around 7–8 h (sleep duration was centered at 7.7 h) (Yoon, 2016; Hirshkowitz et al., 2015). Compared to previous studies, acute sleep deprivation and sleep restriction are not directly comparable to habitual chronic sleep deficiency, and thus comparing studies on experimental sleep restriction and deprivation with studies on habitual sleep duration is inherently challenging. While we found similar relationships between sleep duration and metabolites as previous experimental studies, other observational studies found no association between habitual sleep duration and metabolites (Xiao et al., 2017; Gordon-et al., 2019).

BCAAs have also been explored in other study populations for their relationship with various health conditions, including mental health disorders such as major depression (Ma et al., 2025; Baranyi et al., 2016; Koochakpoor et al., 2021). In these studies, BCAA levels were reported to be associated with major depression, however, the exact relationship remains inconclusive. Patients with major depression showed lower plasma levels of leucine and isoleucine compared to healthy controls (Baranyi et al., 2016). Similarly, upon dietary intake of BCAAs, higher levels of leucine and isoleucine were associated with lower risk of depression and anxiety (Koochakpoor et al., 2021). On the other hand, Zhongxuan and colleagues recently showed that higher plasma levels of leucine and isoleucine associated with higher odds of major depressive disorder (Ma et al., 2025), leaving the direction of the association unclear. Furthermore, an animal study showed that the administration of leucine reduced the entry of neurotoxic kynurenine into the brain and attenuated inflammation-induced depression-like behavior (Walker et al., 2019).

Sleep duration has been linked with depression, where both short (<7h) and long (>9h) sleep were associated with increased odds of depression (Dong et al., 2022). The link between sleep and mental health disorders, such as depression, may be explained though the BCAA levels. In our work, we observed that moderate sleep (sleep centered at 7.7 h, i.e., mean sleep duration) was associated with higher levels of leucine and isoleucine, whereas deviations from moderate levels (i.e, sleep duration longer or shorter than 7.7h) were associated with lower levels of isoleucine and leucine. Together with previous studies on BCAAs and depression, this may suggest that long and short sleep duration may be associated with depression through reduced levels of BCAA (Baranyi et al., 2016; Koochakpoor et al., 2021), including leucine and isoleucine, which in turn may result in increased levels of kynurenine in the brain (Walker et al., 2019).

Sleep timing refers to when sleep is placed within the 24 h of the day (Buysse, 2014), and thus the sleep variables examined in this study; midpoint, social jetlag and chronotype, all reflect features of sleep timing. MSF had strong positive correlations with both social jetlag and chronotype. Of all 3 sleep timing variables, we found MSF to associate with most serum metabolites. Later MSF was associated with metabolites across multiple metabolic pathways, that is, amino acid, lipid and energy pathways. These results are in line with previous observational studies on midpoint and metabolism (Xiao et al., 2017; Gordon-et al., 2019). In line with Xiao et al., we found midpoint to associate with the amino acid and energy pathways (Xiao et al., 2017), and a later midpoint to associate with lower levels of aspartate and creatinine. Lastly, we found that a later MSF was associated with higher levels of LDL/VLDL and adipic acids, and lower levels of albumin lysyl, which is in line with the study by Xiao et al., who found a later midpoint to associate with 29 lipid metabolites, including both positive and negative associations. Therefore, late sleep timing resembles a metabolic profile of dyslipidemia (abnormal levels of lipids), a metabolic signature that in previous studies (Schmid et al., 2015; Davies et al., 2014; Park et al., 2022; Bell et al., 2013; Fritz et al., 2023; Kaneita et al., 2008) also has been linked with sleep deficiency. Social jetlag was associated with the same metabolites (lower aspartate and albumin lysyl) as was found for later MSF. In the cohort, MSF and social jetlag had a moderate positive correlation, thus similar metabolites were expected. Similarly, previous studies have linked social jetlag with amino acid and lipid metabolism (Sládek et al., 2023; Wong et al., 2015). High social jetlag has previously been associated with lipid profiles of dyslipidemia: higher LDL, total cholesterol, triglycerides and lower HDL (Sládek et al., 2023; Wong et al., 2015). Further, high social jetlag has also been linked with abnormal glucose metabolism: higher insulin resistance and fasting insulin levels (Taylor et al., 2016; Wong et al., 2015). Chronotype showed associations with metabolites in the lipid and amino acid pathways; however, at a level of p < 0.01, later chronotype was only associated with lower levels of isobutyrate. The results are partly in line with previous studies, which have shown that extreme morning and evening types were associated with lower HDL levels compared to non-extremes (Sládek et al., 2023). We observed similar associations between later chronotype and dyslipidemia, specifically higher LDL/VLDL, however it did not pass the significance level of p < 0.01.

We investigated multiple sleep variables and found that those representing sleep timing (MSF and social jetlag) were associated with most serum metabolites. Similarly, previous research that investigated both sleep duration and midpoint, found multiple associations with midpoint, but none with sleep duration when comparing with fasting metabolite levels (Xiao et al., 2017; Gordon-et al., 2019). For example, Xiao and colleagues found 64 metabolites to associate with sleep timing out of 329 fasting plasma metabolites, but none with sleep duration (Xiao et al., 2017). Also, Gordon-Dseagu and colleagues found midpoint to associate with 83 metabolites out of 545 known fasting serum metabolites, while none with sleep duration (Gordon-et al., 2019). Further, a recent study examined the relationship between 40 different sleep phenotypes and 768 serum metabolites and reported that the phenotypes on sleep timing were associated with a high number of metabolites (Zhang et al., 2025). Additionally, metabolites are seen to follow a 24-h rhythmicity which is seen to be influenced by behavior and physiological measures (Skene et al., 2018; Isherwood et al., 2017). Skene and colleagues previously measured plasma samples over 24 h in a simulated shift work study and found that 65 metabolites out of 132 showed changes in their 24-h rhythmicity following shift work (Skene et al., 2018). Further, the 24-h rhythmicity of metabolites have also been seen to be altered by BMI and diabetes (Isherwood et al.). Importantly, this indicates other parameters that have the ability to change the 24h rhythmicity of the metabolism (Skene et al., 2018; Isherwood et al. 2017; Potter et al., 2016).

Studies investigating sleep timing with either comprehensive metabolomic profiling or metabolic health factors (Taylor et al., 2016; Sládek et al., 2023), showed that sleep timing was associated with a metabolic profile of hyperglycemia, dyslipidemia and abnormal BCAA metabolism, together with high BMI and insulin resistance (Taylor et al., 2016;Xiao et al., 2017; Sládek et al., 2023; Gordon-et al., 2019; Wong et al., 2015). Collectively, sleep timing (later midpoint and more social jetlag) may induce a metabolic profile that is associated with type 2 diabetes, dyslipidemia and poor cardiometabolic health, specifically through changes in the lipid and amino acid metabolism. Our findings, together with previous studies, underscore the importance of circadian sleep parameters for the human metabolome, and support the hypothesis that sleep timing characterized by late MSF and high social jetlag may be associated with unfavorable metabolic profiles (Xiao et al., 2017; Wong et al., 2015). Together, the findings highlight the clinical importance of sleep timing for metabolic health. The study may further underscore the potential importance of monitoring metabolic health among people working evening and/or night shift, causing a late midpoint and potentially influencing social jetlag. Implementation of metabolic monitoring of shift-workers may aid to attenuate metabolic risk profiles associated with late midpoint and/or high social jetlag. Future studies could also focus on addressing potential interventions targeting specific metabolic risks, such as for instance BCAAs metabolites.

The study has several limitations: First, given the sample size and the exploratory nature of the study, correction for multiple comparisons was not performed. However, we applied a more conservative significance level (*p<0.01*) together with robust linear regressions. Second, this is a cross-sectional study and thus no causality can be inferred between the sleep variables and the metabolites. Third, this study relied on self-reported sleep measures. Future research could benefit from complementing with objective sleep measures, such as actigraphy over a longer period, to capture an average of habitual sleep variables. In contrast, this study has several strengths: First, unlike most previous studies, we investigated sleep and serum metabolome from a multidimensional sleep approach covering four sleep health dimensions. Therefore, this study works in agreement with studying sleep health as more than the absence of sleep disorders and sleep deficiency, and instead looks at how several dimensions of sleep relate to the human metabolome, offering great implications for sleep health promotion and future intervention studies (Buysse, 2014). Second, all blood samples for metabolomic profiling were collected at 8–10 a.m. under fasting conditions since 8 p.m. the day before, which prevented clock time and eating patterns to affect data variance. However, results may have differed had we adjusted to the individual's internal metabolic rhythm, and this may be considered for future similar research (Skene et al., 2018). Third, this study investigated healthy individuals without sleep disorders, allowing us to generalize the findings to healthy adults. Lastly, it examined the link between sleep and metabolism for people of both sexes in a large age span including people aged 20–79 years. Therefore, this study provides important new knowledge to the literature on sleep and metabolomic profiling (Davies et al., 2014; Park et al., 2022; Bell et al., 2013), especially as previous studies had primarily focused on young and middle-aged adults, and sometimes only females or males.

## 5 Conclusion

This study showed that sleep duration, given a U-Shaped representation, and sleep timing (MSF, social jetlag and chronotype) are associated with serum metabolites in an observational cohort of healthy adults. The strongest associations between sleep and metabolism were found for MSF and social jetlag, with metabolites spanning multiple metabolic pathways (notably the amino acid and lipid pathways). To further understand the relationship between sleep and serum metabolome, future studies should examine these associations in larger samples, include objective sleep measures and metabolic health outcomes such as insulin resistance, diabetes and cardiovascular disease.

## CRediT authorship contribution statement

**Charlotte Sørensen:** Writing – review & editing, Writing – original draft, Visualization, Software, Project administration, Methodology, Investigation, Formal analysis, Data curation. **Ilona Dudka:** Writing – review & editing, Writing – original draft, Supervision, Methodology, Investigation, Formal analysis, Conceptualization. **Ana Virel:** Writing – review & editing, Writing – original draft, Supervision, Methodology, Investigation, Conceptualization. **Ingemar Kåreholt:** Writing – review & editing, Supervision, Investigation. **Leonie JT. Balter:** Writing – review & editing, Supervision, Methodology, Investigation, Conceptualization. **John Axelsson:** Writing – review & editing, Supervision, Methodology, Investigation, Conceptualization. **Grégoria Kalpouzos:** Writing – review & editing, Supervision, Resources, Project administration, Methodology, Investigation, Funding acquisition, Data curation, Conceptualization. **Shireen Sindi:** Writing – review & editing, Supervision, Resources, Project administration, Methodology, Investigation, Funding acquisition, Conceptualization.

## Disclosure statement

*Financial disclosure*: none.

## Funding

This work was supported by grants from the Swedish Research Council to Grégoria Kalpouzos (grants 2014-00940, 2018-01327, and 2021–02338), Karolinska Institutet to Grégoria Kalpouzos (grant 2018-01901), and Strategic Neuroscience Program Umeå University-Karolinska Institutet (StratNeuro) to Ana Virel. Shireen Sindi is supported by Swedish Research Council (Dnr: 2020–02325), Alzheimerfonden, The Rut and Arvid Wolff Memorial Foundation, The Foundation for Geriatric Diseases at Karolinska Institutet, Erik Rönnbergs Stipend—Riksbankens Jubileumsfond, Loo and Hans Osterman Foundation for Medical Research.

## Declaration of competing interest

The authors declare that they have no known competing financial interests or personal relationships that could have appeared to influence the work reported in this paper.

## Data Availability

The data that support the findings of this study are available from the corresponding author upon reasonable request.
