# Supplementary material for: Sleep health associations with serum metabolites in healthy adults
Source: Brain Behav Immun Health. 2025 Jul 4;48:101050. doi: 10.1016/j.bbih.2025.101050 (PMC12281066; doi:10.1016/j.bbih.2025.101050)

## Supplementary material

**List of titles and captions**

Table S1

- Title: Full list of serum metabolites, their standard deviation and which major metabolic pathway they belong to.

Figure S1

- Title: Associations between sleep variables and serum metabolites
- Caption: A graphical representation of the serum metabolites that were associated with one or more sleep variables (color coded). All associations shown here were significant at p<0.05, and those with ** were significant at the threshold p<0.01. A positive association (higher metabolite level) is indicated with a + and a negative association (lower metabolite level) is associated with -.

Figure S2

- Title: Distribution of weighted mean sleep duration of the IronAge cohort.
- Caption: The dotted blue vertical lines represent the recommended sleep duration for the cohort including young adults to older adults.

**Table S1**

| **Metabolite** | **Standard deviation** | **Major metabolic pathway** |
| --- | --- | --- |
| Formate | 0.009 | Amino acid |
| Phenylalanine | 0.058 | Amino acid |
| 1-Methylhistidine | 0.013 | Amino acid |
| Tyrosine | 0.018 | Amino acid |
| Threonine | 0.136 | Amino acid |
| Serine | 0.082 | Amino acid |
| Glycine | 0.096 | Amino acid |
| Proline | 0.035 | Amino acid |
| Ornithine | 0.018 | Amino acid |
| Glutamine | 0.200 | Amino acid |
| Glutathione | 0.012 | Amino acid |
| Arginine | 0.031 | Amino acid |
| Aspartate | 0.069 | Amino acid |
| Glutamate | 0.031 | Amino acid |
| Valine | 0.114 | Amino acid |
| Isoleucine | 0.022 | Amino acid |
| Leucine | 0.126 | Amino acid |
| Alanine | 0.146 | Amino acid |
| Methionine | 0.021 | Amino acid |
| 3-Methyl-2-oxovalerate | 0.008 | Amino acid |
| Unsaturated_lipids (–CH=CH–) | 0.540 | Lipid |
| Lipids (CH_2_–C=C) | 0.228 | Lipid |
| Lipids (−CH_2_–C=O) | 0.554 | Lipid |
| Lipids (–CH_3_) (mainly_LDL/VLDL) | 1.583 | Lipid |
| Lipids (CH_2_)_n_ (mainly LDL/VLDL) | 0.441 | Lipid |
| Lipids (=CH–CH_2_–CH=) | 4.127 | Lipid |
| Glycerol_of_lipids | 0.096 | Lipid |
| Albumin_lysyl | 0.045 | Lipid |
| Acetyl_signals_from_glycoproteins | 0.271 | Lipid |
| Adipic_acid | 0.797 | Lipid |
| Choline | 0.041 | Lipid |
| sn-Glycero-3-phosphocholine | 0.047 | Lipid |
| Phosphocholine/sn-Glycero-3-phosphocholine | 1.050 | Lipid |
| Glycerol | 0.037 | Lipid |
| 3-Hydroxybutyrate | 0.064 | Lipid |
| Cholesterol | 0.008 | Lipid |
| Acetoacetate | 0.095 | Lipid |
| α-Glucose | 0.253 | Carbohydrate |
| α&β-Glucose | 0.611 | Carbohydrate |
| Scyllo-inositol | 0.154 | Carbohydrate |
| Myo-inositol | 0.027 | Carbohydrate |
| Lactate | 0.354 | Carbohydrate |
| Pyruvate | 0.042 | Carbohydrate |
| Creatine | 0.024 | Energy |
| Creatinine | 0.019 | Energy |
| Citrate | 0.034 | Energy |
| Malonate | 0.017 | Energy |
| Acetate | 0.057 | Energy |
| Isobutyrate | 0.010 | Gut microbiota |
| Dimethyl sulfone | 1.563 | Gut microbiota |
| 2,3-Butanediol | 0.014 | Gut microbiota |

**Figure S1**


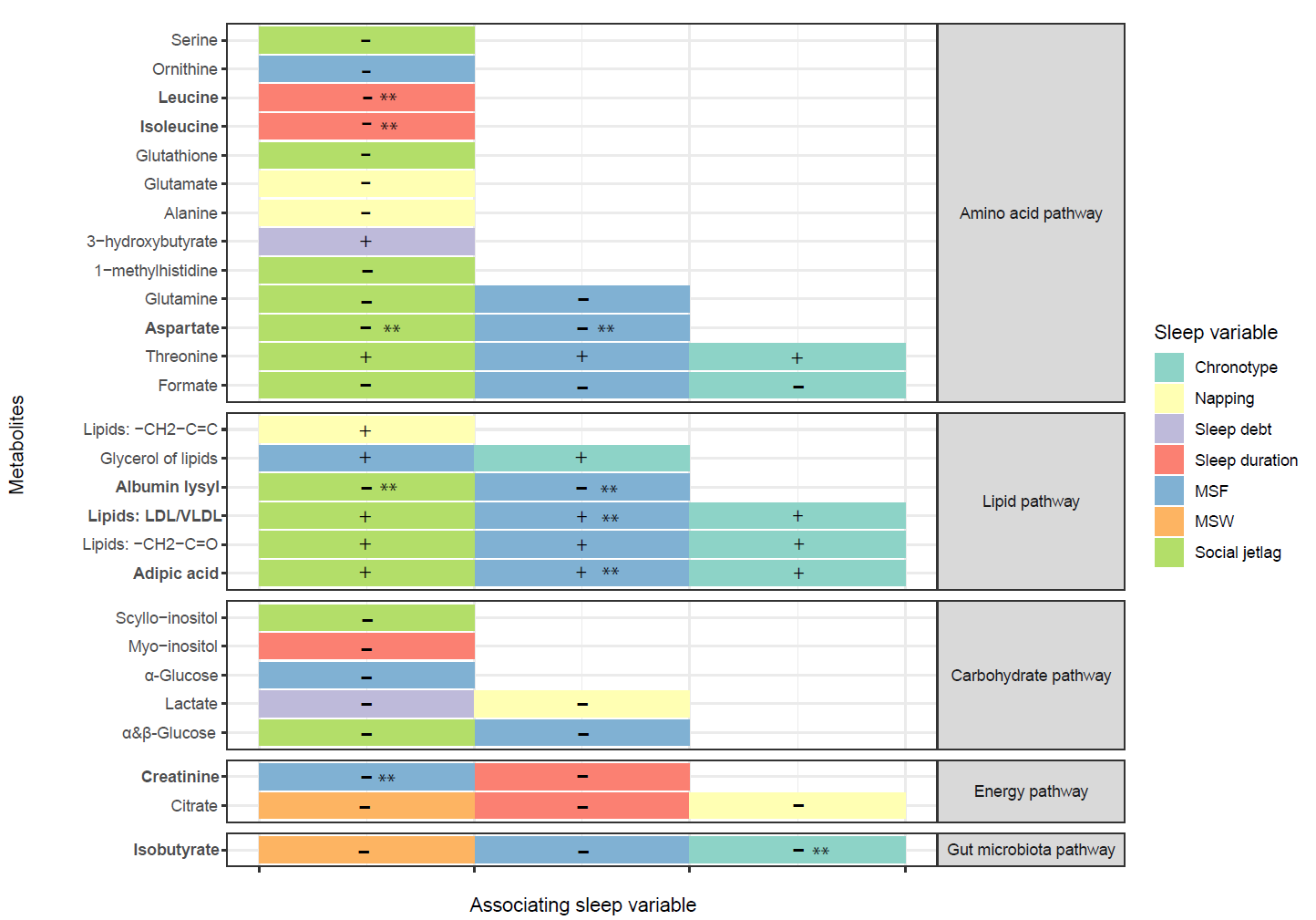


**Figure S2**


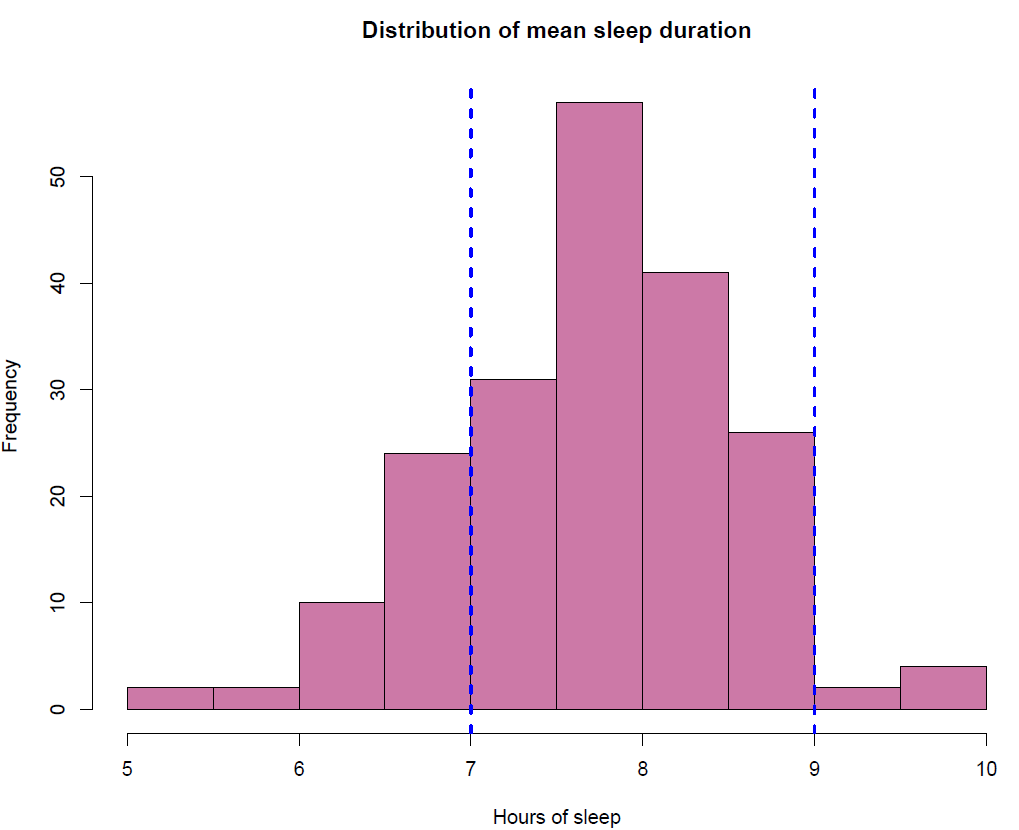

Supplement: Multimedia component 1 [file mmc1.docx]
